# Up-Regulated MicroRNA-27b Promotes Adipocyte Differentiation via Induction of Acyl-CoA Thioesterase 2 Expression

**DOI:** 10.1155/2019/2916243

**Published:** 2019-12-10

**Authors:** Yuka Murata, Takashi Yamashiro, Takaomi Kessoku, Israt Jahan, Haruki Usuda, Tetsuya Tanaka, Takayuki Okamoto, Atsushi Nakajima, Koichiro Wada

**Affiliations:** ^1^Department of Orthodontics and Dentofacial Orthopedics, Graduate School of Dentistry, Osaka University, Suita, 565-0871, Japan; ^2^Department of Gastroenterology and Hepatology, Yokohama City University School of Medicine, Yokohama, 236-0004, Japan; ^3^Department of Pharmacology, Shimane University Faculty of Medicine, Izumo, 693-8501, Japan

## Abstract

Nonalcoholic fatty liver disease (NAFLD) is characterized by a spectrum of liver pathologies, from simple steatosis to steatohepatitis. Recent studies have increasingly noted the aberrant expression of microRNAs closely related to NAFLD pathologies. We have previously shown the presence of increased levels of microRNA-27b (miR-27b) in patients with NAFLD. In this study, we investigated the role of miR-27b in NAFLD by examining the impact of up-regulated miR-27b on the differentiation of preadipocytes into mature adipocytes. We found that miR-27b-3p remarkably enhances the adipocyte differentiation of 3T3-L1 cells associated with lipid accumulation and intracellular triglyceride contents. Furthermore, we have demonstrated not only that miR-27b-3p induces acyl-CoA thioesterase 2 (ACOT2) expression in 3T3-L1 cells, but also that the knockdown of ACOT2 suppresses lipid accumulation and adipocyte differentiation in both the presence and absence of miR-27b-3p treatment. Our data strongly suggest that the miR-27b-ACOT2 axis is an important pathway in adipocyte differentiation and may play a role in the pathogenesis of NAFLD.

## 1. Introduction

Nonalcoholic fatty liver disease (NAFLD), the most common cause of chronic liver disease, is characterized as a spectrum of liver disease ranging from simple steatosis to nonalcoholic steatohepatitis (NASH), which is the most severe form of NAFLD and involves significant hepatocellular injury, fibrosis, and inflammation [1]. NAFLD is associated with obesity, type 2 diabetes mellitus, hyperlipidemia, and insulin resistance, and is closely related to increased cardiovascular events and hepatic disease-related mortality [2]. Moreover, NASH frequently progresses to liver cirrhosis and hepatocellular carcinoma [3]. Adipocyte differentiation from preadipocyte is a prerequisite for the onset of steatosis and includes lipid accumulation and enlargement of liver tissue in the initial stages of NAFLD [4]. Thus, blockade of aberrant adipocyte differentiation leading to lipid deposition in liver tissue is recognized as an effective approach for preventing the development of NAFLD.

Recently, microRNAs (miRNAs) have been touted as a therapeutic target and specific biomarker not only for NAFLD, but also for several cancers and hepatitis C [5]. miRNAs are small (18–25 nucleotides) noncoding RNAs [6] that target the 3′-untranslated regions of their target mRNAs in order to regulate gene expression [7, 8]. miRNAs act in the post-transcriptional regulation of gene expression through inhibition of translation or mRNA degradation [8]. miRNAs are constitutively expressed as one group of regulators in a number of biologic processes [8]. Several studies have analyzed differential miRNA expression in patients with NAFLD and have identified a number of miRNAs at increased or decreased levels during the development of NAFLD and NASH [5, 9].

Previous studies have shown that miRNAs and their regulatory networks are implicated in the pathogenesis of NAFLD and NASH. Reduced expression of miR-122 was observed in hepatic tissues of NAFLD and NASH [9]. In contrast, miR-122 expression was elevated in the serum or blood of NAFLD patients [10]. Knockout of miR-122 expression facilitated triglycerides (TGs) accumulation and hepatic steatosis that progressed to NASH and fibrosis [11]. In addition, miR-34a was up-regulated in tissue and serum of NAFLD patients, and induced down-regulation of NAFLD related genes leading to TGs accumulation and steatosis [9]. Moreover, a number of miRNAs and their target genes has been identified in NAFLD and NASH patients suggesting that the dyscoordination of coding–noncoding RNA regulatory network is important trigger of NAFLD pathogenesis.

In our previous studies, we also compared alterations in miRNA expression in the liver biopsy samples of NASH patients against healthy subjects via extensive miRNA microarray analysis [12, 13]. This result showed reduced expression of miR-122 in liver tissues of NAFLD and NASH patients as well as previous reports. Among altered miRNAs in patients, we found that miR-27b is remarkedly increased in liver tissue and serum from the early phase of NAFLD to severe NASH [12, 13]. It has been reported that miR-27b is involved in cell proliferation, differentiation, migration, invasion, metastasis, multi-drug resistance in various tumor cells [14–18]. However, the exact role of miR-27b in NAFLD pathogenesis, and in particular its relationship to adipocyte differentiation, remains unclear.

In this study, we have investigated the role of miR-27b in 3T3-L1 cell differentiation into adipocytes and other potential mechanisms. We found that miR-27b functionally accelerated mature adipocyte differentiation and excessive lipid accumulation through the induction of acyl-CoA thioesterase 2 (ACOT2) expression.

## 2. Materials and Methods

### 2.1. Cell Culture and Induction of Adipocyte Differentiation

Mouse 3T3-L1 preadipocytes obtained from Primary Cell (Sapporo, Japan) are commonly used to study in vitro adipocyte differentiation. We employed 3T3-L1 cells and induced adipocyte differentiation using a previously described method [19, 20]. Briefly, 3T3-L1 cells were cultured in Dulbecco's modified Eagle's medium (DMEM, Sigma Aldrich, Tokyo, Japan) supplemented with 10% fetal bovine serum (Equitech-Bio, Taxas, USA), 1% MEM nonessential amino acids (Nacalai tesque, Kyoto, Japan), 100 IU/mL penicillin and 0.1 mg/mL streptomycin (Nacalai tesque). Three days after reaching confluency (day 0), the medium was replaced with differentiation medium containing 150 nM insulin (INS, Sigma Aldrich), 1 *μ*M dexamethasone (DEX, Sigma Aldrich), 100 *μ*M 3-Isobutyl-1-methylxanthine (IBMX, Sigma Aldrich) and 1 *μ*M Rosiglitazone (ROSI, GlaxoSmithKline, Tokyo, Japan). The differentiation medium was changed every 4 days until analysis (day 6).

### 2.2. Evaluation of Lipid Accumulation and Adipocyte Differentiation

Lipid accumulation in adipocytes was visualized by Oil red O staining. Cells stained by Oil red O were observed via microscopy. The amount of triglyceride (TG), an index of lipid accumulation, was quantitatively measured by using a Triglyceride E-test Wako kit (Wako Pure Chemicals, Tokyo, Japan). The amount of TG was normalized by the protein amount.

### 2.3. Transfection of miR-27b-3p into 3T3-L1 Cells

Synthesized mimic miR-27b-3p (mature miRNA sequence: AGA GCU UAG CUG AUU GGU GAA C) or negative control (miRNA mimic Negative control) was purchased from Life Technologies (Tokyo, Japan). Negative control or miR-27b-3p was transfected by Lipofectamine RNAiMAX transfection reagent (Life Technologies) into 3T3-L1 cells at a final concentration of 20 nM. Transfection of negative control or miR-27b-3p was performed at day 0 and day 3, respectively. After transfection for 24 hours, transfection medium was replaced with differentiation medium. Adipocyte differentiation and lipid accumulation in cells were assessed at day 6 by Oil red O staining and a TG assay. The cell number at day 6 was counted by using a Countess Automated Cell Counter (Thermo Fisher Scientific, Tokyo, Japan).

### 2.4. DNA Microarray Analysis of miR-27b-Transfected 3T3-L1 Cells

After a second transfection of miR-27b-3p or negative control for 24 hours, total RNA was purified by using TRizol reagents (Thermo Fisher Scientific) and applied for Kurabo-DNA microarray analysis (KURABO Bio-Medical department, Osaka, Japan). The relative expression level of a given mRNA was calculated by comparing the signal intensities of valid spots throughout the microarray experiments. All analyzed data were scaled by global normalization and were expressed as the ratio of the signal of cells. We selected genes that showed more than a 1.7-fold induction or less than a 0.6-fold repression according to the previously described methods [20, 21]. Gene ontology (GO) enrichment analysis of differentially expressed genes was assessed with PANTHER (http://www.pantherdb.org/) and then focused on genes in relation to adipocyte differentiation and lipid metabolisms by referring to the gene's function.

### 2.5. Detection of ACOT2 Protein Expression by Western Blotting

After transfection and differentiation, 3T3-L1 cells were harvested in lysis buffer with 0.1% of a proteinase inhibitor cocktail (Promega, Tokyo, Japan). Equal amounts of protein were subjected to SDS-PAGE and analyzed by Western blotting. ACOT2 protein was detected using anti-ACOT2 polyclonal antibody (Proteintech, Chicago, IL; 1 : 1000) and peroxidase-conjugated anti-rabbit IgG (Bio-Rad Laboratories, Richmond, CA; 1 : 2000). Glyceraldehyde-3-phosphate dehydrogenase (GAPDH) protein was detected using anti-GAPDH antibody (Trevigen, Gaithersburg, MD) and peroxidase-conjugated anti-rabbit IgG. Blots were developed by chemiluminescence (Immobilon Western, Millipore, Billerica, MA).

### 2.6. Knockdown of ACOT2 Expression in 3T3-L1 Cells by RNA Interference

Specific small interfering RNAs (siRNAs) against ACOT2 mRNA were designed by Stealth RNAi-siRNA system (Life Technologies). The sense and antisense sequences of ACOT2-siRNA were 5′-UGG UGG CCU CGU CUU UCG CUG UCC U-3′, and 5′-AGG ACA GCG AAA GAC GAG GCC ACC A-3′, respectively. ACOT2-siRNAs or negative control siRNAs (Stealth RNAi Negative Control Duplexes) were transfected by Lipofectamine RNAiMAX at a final concentration of 10 nM siRNA. After transfection for 24 hours, the medium was replaced with differentiation medium to induce differentiation. Transfection of siRNAs was performed at day 0 and day 3. As combination treatment with miR-27b-3p and ACOT2-siRNA, both transfection-reagents, was applied to cultured cells at the same time. After differentiation, lipid accumulation and cell numbers were evaluated.

### 2.7. Statistical Analysis

Results are expressed as the mean ± SEM. Statistical comparisons of TG contents and cell proliferation between control-miR and miR-27b-3p were performed using the Student's *t*-test. Statistical comparisons of TG contents between control-miR, miR-27b-3p, and combination with miR-27b-3p and ACOT siRNA were performed using Scheffe's method after analysis of variance (ANOVA). The results were considered significantly different at *P* < 0.05.

## 3. Results

### 3.1. MIR-27b-3p Increased Lipid Accumulation and TG Contents in 3T3-L1 Cells after Adipocyte Differentiation

**Figure 1:**
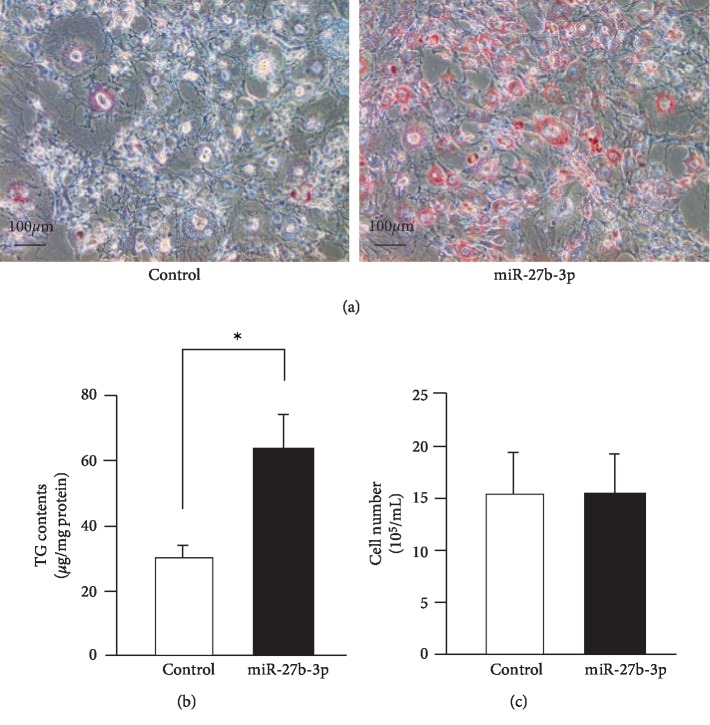
miR-27b increases lipid accumulation and adipocyte differentiation. (a) Oil red O staining of 3T3-L1 cells after adipocyte differentiation. At day 0 and day 3, control-miR (left panel) and miR-27b (right panel) were transfected into 3T3-L1 cells. Representative data from three different images are shown. Scale bars: 100 *μ*m. (b) Intracellularly TG contents of control-miR and miR-27b transfected 3T3-L1 cells were measured. Each column represents the mean ± SEM from 6 independent experiments. ^∗^Significantly difference than control-miR by Student's *t*-test (*P* < 0.05). (c) Numbers of control-miR or miR-27b-transfected 3T3-L1 cells after adipocyte differentiation. Each column represents the mean ± SEM from 3 independent experiments.

Adipocyte differentiation is primarily controlled by CCAAT/enhancer binding protein (C/EBP) and PPAR*γ* at the transcriptional level [22, 23]. In the experimental model, DEX, IBMX, INS, and ROSI were used as inducing agents of adipocyte differentiation. We confirmed adipocyte differentiation after these stimulation processes (data not shown). In order to examine the effect of miR-27b on adipocyte differentiation, we transfected mimetic miR-27b-3p into 3T3-L1 cells and evaluated lipid deposition in the intracellular space following differentiation. Transfection of miR-27b-3p remarkedly increased lipid deposition in 3T3-L1 cells compared to transfection of control-miR (Figure 1(a)). TG contents, which represent the index of mature adipocyte, were also significantly higher in miR-27b-3p-transfected cells, more so than in control-miR-transfected cells. To investigate the effect of miR-27b on cell proliferation, we determined the number of miR-27b-3p-transfected cells after differentiation (Figure 1(b)). In contrast to control-miR-transfected cells, miR-27b-3p did not influence the total cell number during our experimental period and conditions (Figure 1(c)). These results, which show that miR-27b enhanced adipocyte differentiation of 3T3-L1 cells, suggest that miR-27b is involved in the regulation of adipocyte differentiation- or lipid metabolism-related gene expression.

### 3.2. Identification of the MIR-27b-3p-Regulated Genes Responsible for Adipocyte Differentiation and Lipid Metabolism

**Figure 2:**
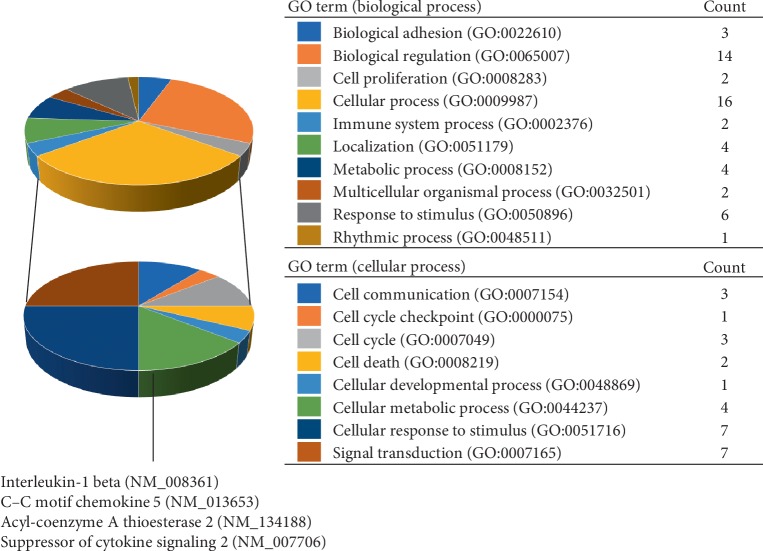
Functional GO enrichment analysis showed a significant phenotypic difference in differentiated 3T3-L1 cells after miR-27b-3p transfection. The microarray data was assessed for biological process function using PANTHER. Enriched biological functions (biological process) of selected up-regulated and down-regulated genes in Tables 1 and 2 (upper pie chart) and the gene counts involved in each process were shown. Subcategories for cellular process were shown in lower pie chart.

**Figure 3:**
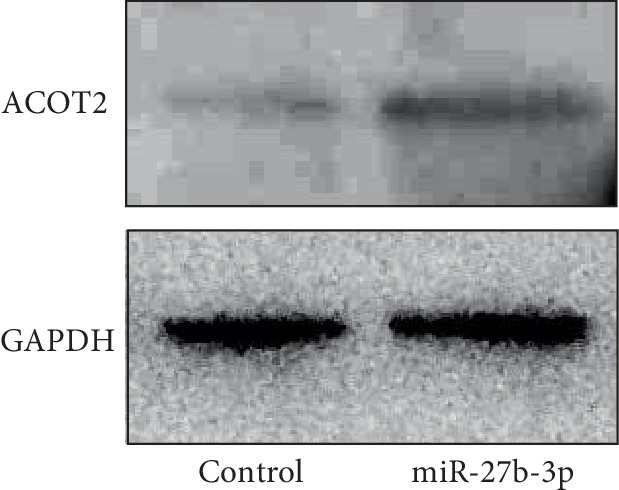
miR-27b induces ACOT2 expression during adipocyte differentiation. Immunoblotting of ACOT2 in control-miR and miR-27b transfected 3T3-L1 cells after adipocyte differentiation. GAPDH was used as an internal standard. Representative data from three different images are shown.

To determine the target genes of miR-27b-3p in 3T3-L1 cells during adipocyte differentiation, we carried out comprehensive DNA microarray analysis that compared the gene expression patterns of differentiated 3T3-L1 cells transfected with either miR-27b-3p or control miR. Of note, miR-27b-3p significantly up-regulated adipocyte differentiation- and lipid metabolism-related genes such as Acyl-CoA thioesterase 2 (ACOT2), prolactin family 2, subfamily c, member 5 (PRL2C5), epiregulin (EREG), cyclin D1 (CCND1) and krüppel-like transcription factor 4 (KLF4) (Table 1). In contrast, miR-27b-3p down-regulated aldo-keto reductase family 1, member B7 (AKR1B7), glutathione S-transferase, alpha 4 (GSTA4), endothelin receptor type A (EDNRA), and signal transducer and activator of transcription 1 (STAT1) (Table 2). GO enrichment analysis of the microarray results revealed a general trend showing that miR-27b-3p regulated a group of genes during the biological regulation and cellular process (Figure 2). In the category of cellular process, we identified cellular metabolic process-related genes including interleukin-1*β* (NM_008361), C–C motif chemokine 5 (NM_013653), ACOT2 (NM_134188), suppressor of cytokine signaling 2 (NM_007706). Notably, although the basal signal intensity of ACOT2 was higher than the other genes, the altered ratio of ACOT2 mRNA was remarkedly larger. To validate ACOT2 as a responsive gene of miR-27b-3p, we confirmed the increased ACOT2 protein expression in miR-27b-3p-transfected 3T3-L1 cells after differentiation (Figure 3). In summary, we focused on ACOT2, which is known as an enzyme that facilitates the hydrolysis of CoA and free fatty acids.

### 3.3. ACOT2 Contributes to MIR-27b-Driven Adipocyte Differentiation

**Figure 4:**
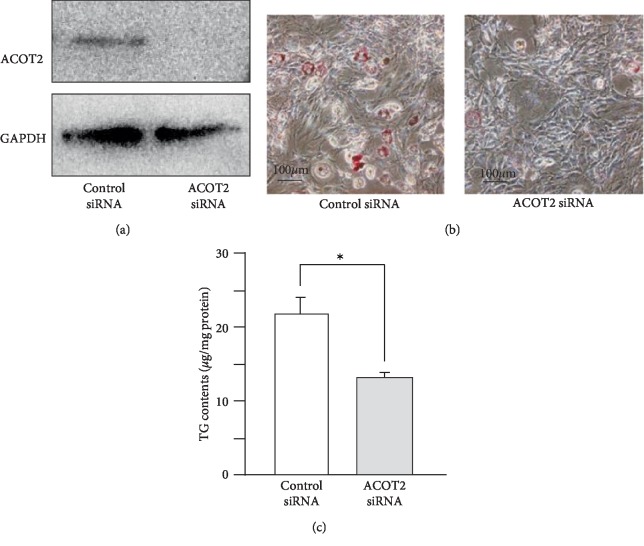
Knockdown of ACOT2 expression suppresses adipocyte differentiation. (a) Immunoblotting of ACOT2 in control-siRNA and ACOT2-siRNA transfected 3T3-L1 cells after adipocyte differentiation. Representative data from three different images are shown. (b) Oil red O staining of control siRNA and ACOT2 siRNA transfected 3T3-L1 cells after adipocyte differentiation. Representative data from three different images are shown. Scale bars: 100 *μ*m. (c) Intracellularly TG contents of control siRNA and ACOT2 siRNA transfected 3T3-L1 cells were measured. Each column represents the mean ± SEM from 6 independent experiments. ^∗^Significantly difference than control siRNA by Student's *t*-test (*P* < 0.05).

**Figure 5:**
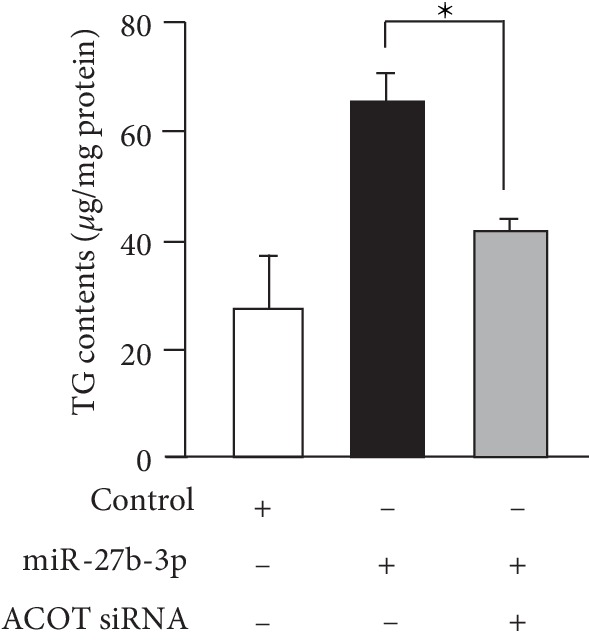
ACOT2 knockdown cancelled miR-27b-driven adipocyte differentiation. Intracellular TG contents of 3T3-L1 cells treated with a combination of miR-27b and ACOT2-siRNA were measured. Each column represents the mean ± SEM from 5 to 7 independent experiments. ^∗^Significantly difference than the combination of miR-27b and ACOT2-siRNA group by Scheffe's method after ANOVA (*P* < 0.05).

Next, we designed an ACOT2-specific siRNA for the knockdown of ACOT2 expression. Treatment with ACOT2-specific siRNAs successfully suppressed expression of ACOT2 protein (Figure 4(a)). We examined the impact of ACOT2 on lipid accumulation and adipocyte differentiation. ACOT2-specific siRNAs significantly reduced lipid accumulation, as well as the amount of TG contents in 3T3-L1 cells after differentiation compared to negative control siRNAs (Figures 4(b) and 4(c)). Furthermore, in order to investigate whether miR-27b-3p-mediated the up-regulation of ACOT2 to promote adipocyte differentiation, we transfected 3T3-L1 cells with both miR-27b-3p and ACOT2-siRNA and then evaluated adipocyte differentiation. As shown in Figure 5, the combined treatment of miR-27b with the ACOT2-siRNA group significantly reduced TG contents in 3T3-L1 cells after differentiation compared to that treated with miR-27b-3p alone. In addition, ACOT2-siRNA cancelled the increase in TG contents induced by miR-27b-3p treatment and recovered TG contents to the same level as the control group. These results clearly demonstrate that ACOT2 predominantly contributes to the promotion of adipocyte differentiation by up-regulating miR-27b.

## 4. Discussion

Several studies have indicated that miR-27b plays an important role in lipid metabolism and adipogenesis [24, 25]. It has been reported that miR-27b is down-regulated in in vitro cultured adipocytes and adipose tissue in obesity-related models and that miR-27b impairs adipocyte differentiation in 3T3-L1 cells and human adipose-derived mesenchymal stem cells via its associations with PPAR*γ* and C/EBP*α* [24–27]. In zebrafish, depletion of miR-27b has shown increases in PPAR*γ*, C/EBP*α*, and SREBP-1c, resulting in the enhancement of adipocyte hyperplasia [28]. In addition to these regulators, miR-27b negatively regulates lysyl oxidase and other genes involved in adipocyte differentiation [29]. Thus, these studies suggest that miR-27b negatively regulates NAFLD pathogenesis. Surprisingly, we have nevertheless found that miR-27b was upregulated in NAFLD biopsy samples from both early and late stages of NASH. In agreement with our own results, an increase in miR-27b was observed in fat tissue and under hypoxic conditions [25]. In summary, alterations in miR-27b expression in NAFLD remain controversial.

In addition to miR-27b, another miR-27 family gene miR-27a has been known as a negative regulator of adipogenesis. The expression of miR-27a was down-regulated upon adipogenic differentiation of 3T3-L1 preadipocytes. Moreover, miR-27a suppressed lipid accumulation in rat hepatic stellate cells and human hepatoma cells by targeting retinoid X receptor *α* [30, 31] and impair adipocyte differentiation by targeting PPAR*γ* [32]. Together, these finding suggest a potential role of miR-27a in multiple metabolic pathways during NAFLD pathogenesis. However, our miRNA microarray data showed up-regulation of miR-27a as well as miR-27b. Although the ratio of hold increased of miR-27a was smaller than that of miR-27b, the up-regulation of miR-27a was observed in from onset of NAFLD to late stages of NASH. Several discrepancies about specific miRNA expression in NAFLD has been reported. The mechanisms remain unclear, but it is thought that the co-regulatory networks of noncoding RNAs including miRNAs, long noncoding RNAs, circular RNAs, P-element-induced wimpy testis (PIWI)-interacting RNAs, and enhancer RNAs may be able to explain the discrepancies [33]. As current reports suggested the potential synergistic effect of co-regulation and interactions between these noncoding RNAs, the co-regulatory network may expand the understanding of the molecular regulation and its complexity in NAFLD development and progression [34].

Several pathways by which the amount of miRNA in a tissue is increased have been proposed. In addition to the biosynthesis of miRNA in cells, recent studies have indicated the existence of circulating miRNAs and the intercellular transfer of miRNAs from distinct donor cells to recipient cells via blood circulation. Exosomes are a known miRNA supply source from donor cells. Exosomes are nano-scale extracellular vehicles (EVs) released by various types of cells [35]. EVs contain a variety of cargos including proteins, mRNAs and miRNAs in donor cells, and are involved in the cell-cell communications with recipient target cells by transferring them [36]. Notably, this paracrine signaling of miRNA plays an important role in cancer metastasis [37]. In this line, increases of miR-27b in the liver tissue of NAFLD patients might be explained by both biogenesis in hepatic cells and paracrine regulation. In our previous study, miR-27b was found to be up-regulated in both liver tissue and serum in patients. These suggested the possibility that miR-27b in EVs is provided by distant donor cells and accumulates in the liver tissue of NAFLD patients. This pathway might explain the up-regulation of miR-27b in NAFLD patients.

In this study, we performed DNA microarray analysis and identified up-regulated and down-regulated mRNA via the transfection of miR-27b-3p in 3T3-L1 cells. However, PPAR*γ*, C/EBP*α*, and SREBP-1c mRNAs did not exhibit significant changes in our microarray data. GO analysis was utilized to identify the putative candidate genes of miR-27b-3p implicated in lipid metabolism and adipocyte differentiation. Among genes altered by miR-27b-3p treatment, we focused on ACOT2, which is an enzyme of the ACOT family involved in the hydrolysis of fatty acyl-CoAs into free fatty acids and CoA-SH [38, 39]. ACOT2 is localized to the mitochondrial matrix and maintains an adequate rate of *β*-oxidation by retaining the same level of coenzymes used in the tricarboxylic acid (TCA) cycle and in β-oxidation itself. ACOT2 expression in the mitochondria increased following adipocyte differentiation [38, 39]. In this study, we have shown that miR-27b increased the expression of ACOT2, and that the increase in ACOT2 played an important role in lipid accumulation and preadipocyte differentiation. Moreover, knockdown of ACOT2 mRNA and protein by a specific siRNA clearly showed the suppression of adipocyte differentiation. These results therefore plainly indicate that the miR-27b-ACOT2 axis promotes lipid accumulation and adipocyte differentiation, process which result in NAFLD pathogenesis.

Several studies have shown that miR-27b regulates not only adipogenesis, but also cell proliferation, angiogenesis, and tumor development [14, 15, 40, 41]. Nonetheless, the role of miR-27b has not been fully elucidated. For example, up-regulated miR-27b in the serum of Kawasaki disease patients enhanced endothelial cell proliferation and migration by targeting Smad7 [40]. In contrast, down-regulated miR-27b promoted the activation of adenosine monophosphate-activated protein kinase, which induced angiogenic tube formation and the migration of endothelial cells [15]. In addition, miR-27b highly expressed in sheep skeletal muscle directly reduced myostatin mRNA and led to cell proliferation [41]. In contrast, down-regulated miR-27b in oral lichen planus promotes human oral keratinocytes proliferation via increased Polo-like Kinase-2 expression [14]. Moreover, the down-regulation and up-regulation of miR-27b promotes breast cancer metastasis and gastric cancer, respectively. These studies suggest that miR-27b has different expression pattern and functions depending on the cell type, biological process, diseases, and experimental conditions.

Our study provides the first evidence that up-regulated miR-27b in NAFLD patients promotes adipogenesis and lipid deposition in 3T3-L1 cells during adipocyte differentiation. A similar experiment showed inconsistent results; the authors found that miR-27b in 3T3-L1 cells stimulated by DEX, IBMX, and INS became down-regulated and that exogenous miR-27b reduced lipid accumulation [25]. In our model, we stimulated 3T3-L1 cells with DEX, IBMX, INS, and ROSI to achieve adipocyte differentiation. This occurred in response to NAFLD development under these signaling cascades. Notably, PPAR*γ* is a master transcriptional regulator of adipogenesis and lipid metabolism [42]. Indeed, since increased PPAR*γ* expression has been found in steatotic livers, it has been suggested that the role of PPAR*γ* in the activation of lipogenic genes may contribute to the development of steatosis [1]. In agreement with PPAR*γ* activation in the fatty liver of obese human subjects, we stimulated cells with PPAR*γ* and other inducers, which resulted in the enhancement of adipocyte differentiation [19]. Thus, PPAR*γ* activation might be critical for miR-27b expression under PPAR*γ* signaling; however, further studies are required for elucidation.

PPAR*γ* is highly expressed not only in adipose tissue, but also in the liver of NAFLD patients and in experimental models [43, 44]. PPAR*γ* deletion in mouse hepatocytes has been shown to protect against the development of steatosis [45]. Moreover, several studies have shown that PPAR*γ* activation can prevent the progression of hepatic steatosis in murine models [46], and that treatment with the PPAR*γ* agonist rosiglitazone exerts similar effects [47]. The protective effects of PPAR*γ* could be the result of higher insulin sensitivity in adipose tissue and skeletal muscles, leading to a reduction in free fatty acid deposition in the liver [45]. PPAR*γ* has been shown to induce adiponectin, which also contributes to insulin sensitivity, and PPAR*α* expression, in turn leading to further hepatic fatty acid oxidation. Furthermore, PPAR*γ* expression has been shown to promote anti-inflammatory and anti-fibrotic effects in stellate cells, macrophages, and epithelial cells [48]. In addition to adipocyte differentiation, it remains unclear whether miR-27b is involved in such PPAR*γ*-related effects as improvements in insulin sensitivity, anti-inflammatory and anti-fibrotic effects.

## 5. Conclusions

In summary, this study has successfully provided important information about the roles of up-regulated miR-27b in the context of NAFLD pathogenesis. Our result suggests that miR-27b-3p induces ACOT2 expression and that the miR-27b-3p-ACOT2 axis contributes to adipocyte differentiation. Further investigation will be required to clarify the exact mechanisms at work. Nonetheless, miR-27b-ACOT2 axis may potentially play a role in the pathological progression of NAFLD.

## Figures and Tables

**Table 1:** Microarray analysis of gene expression increases on miR-27b transfection.

Gene description	Gene accession	Fold increase
Prolactin family 2, subfamily c, member 5 (Prl2c5)	NM_181852	3.672185
Serum amyloid A 3 (Saa3)	NM_011315	3.367436
CD53 antigen (Cd53)	NM_007651	2.202753
Thrombospondin 1 (Thbs1)	NM_011580	2.166568
Lipopolysaccharide binding protein (Lbp)	NM_008489	2.148915
Connective tissue growth factor (Ctgf)	NM_010217	2.090627
Epiregulin (Ereg)	NM_007950	2.087868
Nuclear receptor subfamily 4, group A, member 1 (Nr4a1)	NM_010444	2.059057
CD55 antigen (Cd55)	NM_010016	2.0581
Rho-guanine nucleotide exchange factor (Rgnef)	NM_012026	2.044236
Retinoic acid receptor responder (tazarotene induced) 1 (Rarres1)	NM_001164763	2.041146
Suppressor of cytokine signaling 2 (Socs2), transcript variant 1	NM_007706	2.03834
ATPase, Na+/K+ transporting, alpha 2 polypeptide (Atp1a2)	NM_178405	2.019371
Adrenomedullin (Adm)	NM_009627	2.005821
Acyl-CoA thioesterase 2 (Acot2), nuclear gene encoding mitochondrial protein	NM_134188	1.973443
CD34 antigen (Cd34), transcript variant 1	NM_001111059	1.938985
Ets variant gene 4 (E1A enhancer binding protein, E1AF) (Etv4)	NM_008815	1.932054
Frizzled homolog 4 (Drosophila) (Fzd4)	NM_008055	1.91573
Creatine kinase, mitochondrial 1, ubiquitous (Ckmt1), nuclear gene encoding mitochondrial protein	NM_009897	1.912341
Cyclin D1 (Ccnd1)	NM_007631	1.910614
Kruppel-like factor 4 (gut) (Klf4)	NM_010637	1.893031
Fibroblast growth factor receptor 3 (Fgfr3), transcript variant 1	NM_008010	1.863915
Inhibitor of DNA binding 2 (Id2)	NM_010496	1.861815
Dual specificity phosphatase 6 (Dusp6)	NM_026268	1.844081
Acid phosphatase 5, tartrate resistant (Acp5), transcript variant 2	NM_001102404	1.842465
Oxidized low density lipoprotein (lectin-like) receptor 1 (Olr1)	NM_138648	1.810867
Suppressor of variegation 3–9 homolog 1 (Drosophila) (Suv39h1)	NM_011514	1.802061
Cell division cycle 6 (Cdc6), transcript variant 1	NM_011799	1.768399
Phosphodiesterase 4B, cAMP specific (Pde4b), transcript variant 1	NM_019840	1.768212
Serum amyloid A 1 (Saa1)	NM_009117	1.762458
Matrix metallopeptidase 1b (interstitial collagenase) (Mmp1b)	NM_032007	1.761906
Proviral integration site 1 (Pim1)	NM_008842	1.741448

**Table 2:** Microarray analysis of gene expression decreases on miR-27b transfection.

Gene description	Gene accession	Fold increase
Natriuretic peptide receptor 1 (Npr1)	NM_008727	0.57428
Dickkopf homolog 2 (*Xenopus laevis*) (Dkk2)	NM_020265	0.572471
Aldo-keto reductase family 1, member B7 (Akr1b7)	NM_009731	0.553844
Glutathione S-transferase, alpha 4 (Gsta4)	NM_010357	0.551271
Interleukin 1 beta (Il1b)	NM_008361	0.543772
Major facilitator superfamily domain containing 2A (Mfsd2a)	NM_029662	0.541809
Chemokine-like receptor 1 (Cmklr1)	NM_008153	0.530848
Lipase, family member K (Lipk), transcript variant 1	NM_001205349	0.530183
Epithelial stromal interaction 1 (breast) (Epsti1), transcript variant a	NM_029495	0.514737
Endothelin receptor type A (Ednra)	NM_010332	0.476219
Chemokine (C–C motif) ligand 5 (Ccl5)	NM_013653	0.469792
Signal transducer and activator of transcription 1 (Stat1), transcript variant 2	NM_009283	0.450286
Epithelial cell adhesion molecule (Epcam)	NM_008532	0.448999
Glutathione S-transferase, alpha 3 (Gsta3), transcript variant 1	NM_001077353	0.422778
Secreted frizzled-related protein 2 (Sfrp2)	NM_009144	0.398325

## Data Availability

All data used to support the findings of this study are included within the article.
